# Fluoxetine regulates eEF2 activity (phosphorylation) via HDAC1 inhibitory mechanism in an LPS-induced mouse model of depression

**DOI:** 10.1186/s12974-021-02091-5

**Published:** 2021-02-01

**Authors:** Weifen Li, Tahir Ali, Chengyou Zheng, Zizhen Liu, Kaiwu He, Fawad Ali Shah, Qingguo Ren, Shafiq Ur Rahman, Ningning Li, Zhi-Jian Yu, Shupeng Li

**Affiliations:** 1 State Key Laboratory of Oncogenomics, School of Chemical Biology and Biotechnology, Peking University Shenzhen Graduate School, Shenzhen, 518055 China; 2 Riphah Institute of Pharmaceutical Sciences, Riphah International University Islamabad, Islamabad, Pakistan; 3 Department of Neurology, Affiliated ZhongDa Hospital, School of Medicine, Southeast University, Nanjing, China; 4 Department of Pharmacy, Shaheed Benazir Bhutto University, Sheringal, Dir, 18000, Pakistan; 5 Tomas Lindahl Nobel Laureate Laboratory, Precision Medicine Research Centre, The Seventh Affiliated Hospital of Sun Yat-sen University, Shenzhen, 518107 China; 6 Department of Infectious Diseases and Shenzhen Key Laboratory for Endogenous Infections, The 6th Affiliated Hospital of Shenzhen University Health Science Center, No 89, Taoyuan Road, Nanshan District, Shenzhen, 518052 China; 7 Campbell Research Institute, Centre for Addiction and Mental Health, Toronto, Ontario Canada; 8 Department of Psychiatry, University of Toronto, Toronto, Ontario Canada

**Keywords:** Fluoxetine, Neuroinflammation, Depression, Synaptogenesis, HDAC1-eEF2

## Abstract

**Background:**

Selective serotonin reuptaker inhibitors, including fluoxetine, are widely studied and prescribed antidepressants, while their exact molecular and cellular mechanism are yet to be defined. We investigated the involvement of HDAC1 and eEF2 in the antidepressant mechanisms of fluoxetine using a lipopolysaccharide (LPS)-induced depression-like behavior model.

**Methods:**

For in vivo analysis, mice were treated with LPS (2 mg/kg BW), fluoxetine (20 mg/kg BW), HDAC1 activator (Exifone: 54 mg/kg BW) and NH125 (1 mg/kg BW). Depressive-like behaviors were confirmed via behavior tests including OFT, FST, SPT, and TST. Cytokines were measured by ELISA while Iba-1 and GFAP expression were determined by immunofluorescence. Further, the desired gene expression was measured by immunoblotting. For in vitro analysis, BV2 cell lines were cultured; treated with LPS, exifone, and fluoxetine; collected; and analyzed.

**Results:**

Mice treated with LPS displayed depression-like behaviors, pronounced neuroinflammation, increased HDAC1 expression, and reduced eEF2 activity, as accompanied by altered synaptogenic factors including BDNF, SNAP25, and PSD95. Fluoxetine treatment exhibited antidepressant effects and ameliorated the molecular changes induced by LPS. Exifone, a selective HDAC1 activator, reversed the antidepressant and anti-inflammatory effects of fluoxetine both in vivo and in vitro, supporting a causing role of HDAC1 in neuroinflammation allied depression. Further molecular mechanisms underlying HDAC1 were explored with NH125, an eEF2K inhibitor, whose treatment reduced immobility time, altered pro-inflammatory cytokines, and NLRP3 expression. Moreover, NH125 treatment enhanced eEF2 and GSK3β activities, BDNF, SNAP25, and PSD95 expression, but had no effects on HDAC1.

**Conclusions:**

Our results showed that the antidepressant effects of fluoxetine may involve HDAC1-eEF2 related neuroinflammation and synaptogenesis.

**Supplementary Information:**

The online version contains supplementary material available at 10.1186/s12974-021-02091-5.

## Introduction

Major depressive disorders (MDD) are common but serious mood disorders, affecting millions of people worldwide [1, 2]. Despite the dramatic increase in antidepressants, present treatments are ineffective to one third of patients [3–5], indicating the urgent need for more reliable and effective antidepressants. Among the complex mechanisms, growing shreds of evidence have supported the involvement of neuroinflammation in the pathophysiology of depression [6–9]. Under stresses such as psychological stimuli and physical illness, the release of inflammatory cytokines and glial cell activation can be dramatic, leading to apoptosis, attenuating neuronal differentiation, and suppressing synaptic transmission and maintenance of long-term potentiation, and finally, result in MDD [7–13]. Thus, the questions arose whether neuroinflammation plays a causative role in the pathophysiology of depression [7, 8, 14] and if the current antidepressants may contribute to the suppression of neuroinflammation [6, 15]. In this regard, we recently proved the antidepressive effects of melatonin via attenuating neuroinflammation and as inflammation allied autophagy impairment [15, 16].

Histone deacetylases (HDACs) are the enzymes that induce the deacetylation of histone protein at lysine residues. Class 1 among the 4 classes of HDACs is the most frequently studied histone modifier and transcriptional repressor [17, 18]. Dysregulation of HDACs leads to deacetylation and acetylation impairment which may be involved in the pathological process of diseases including depression [19–21]. HDACs control chromatin architecture around the genes, which are involved in the pathophysiology of depression and the action mechanism of antidepressants [22, 23]. Likely, HDAC2-mediated H3 acetylation in the nucleus accumbent is involved in depression through long-lasting positive neuronal adaptations [24]. Furthermore, accumulating evidence supports the role of HDACs in innate immunity, which acts both as a positive and negative regulator of Toll-like receptors (TLR) signaling [25, 26]. HDAC deacetylates LPS-acetylated mitogen-activated protein kinase phosphatase (MKP)-1, which then sustains p38 activation and promotes TLR-inducible inflammatory response [27, 28]. However, although preclinical tests show the potential of HDACs inhibitors as antidepressants [21, 24, 29], none of them has been applied in clinical treatment due to the lack of selectivity and risk of serious adverse events [19]. Thus, delineating the etiological role of specific HDAC isoforms would facilitate the application of novel and highly selective HDAC modulators in depression.

Recent evidence indicates that dysregulation of key synaptic protein synthesis and related dendritic and spine complexity underlies the core pathology of depression [30]. Eukaryotic elongation factor 2 (eEF2) is known to be at least partially involved in the peptide-chain elongation process of protein synthesis upon stimulation of diverse stimuli [31]. Also, ketamine failed to induced its antidepressant effects in animals pre-treated with protein synthesis inhibitors, suggesting that eEF2-induced translation possibly driven by BDNF (Brain-derived neurotrophic factor) is important to the antidepressant action of ketamine [32]. Interestingly, in response to stresses including mTORC1 (mammalian target of rapamycin) inhibition, eEF2K can be activated, followed by eEF2 phosphorylation and inhibition, eventually leading to reduced protein translation [33–35]. Thus, enhancing eEF2 activity by antidepressants might be a crucial strategy against depression.

Fluoxetine is a selective serotonin reuptake inhibitor (SSRIs) and has been clinically widely used for depression [36–38]. It is postulated that fluoxetine and other SSRIs modulate serotonin levels at the central nervous system’s synaptic level to regulate mood disorders [39, 40]. Later studies reveal that fluoxetine also exerts neuroprotective [41], anti-cancer [42, 43], and anti-inflammatory effects [41, 44]. Additional mechanistic results demonstrate that independent of serotonin level adjustment, and fluoxetine treatment promotes neuroplasticity via tropomyosin receptor kinase B (TrkB)/BDNF [45] and neurogenesis via glycogen synthase kinases (GSK-3β)/β-catenin signaling pathway [46], which may also contribute to its antidepressant effects. Although previous studies demonstrate HDAC activity involvement in anti-depression [29, 47], and HDAC inhibition can re-boost the antidepressant effect of fluoxetine [48, 49], the detailed link between specific HDAC subtypes and how it contributes to the antidepressant effects of fluoxetine are yet unknown. Here, we demonstrated that HDAC1-eEF2 activation led to increased synaptogenesis, which may underlie the antidepressant effects of fluoxetine. These results may shed further insights into the molecular mechanism of fluoxetine and may provide alternative strategic clues for the HDAC1 inhibitors as novel antidepressants.

## Materials and methods

### Animal and drug treatment

Adult C57BL/6J male mice weighing 25–30 g (12–14 weeks) were purchased from Guangdong Medical Laboratory Animal Center, China. The experimental animals were housed at Laboratory Animal Research Center, Peking University Shenzhen Graduate School, under 12-h light/12 h dark cycle at 18–22 °C and had free access to diet and tap water throughout the study. The experimental procedures were set in such a way to minimize mice suffering. All experimental procedures were carried out according to the protocols approved by the Institutional Animal Care and Use Committee of Peking University Shenzhen Graduate School.

The study was conducted into three experiments.

**Fig. 1:**
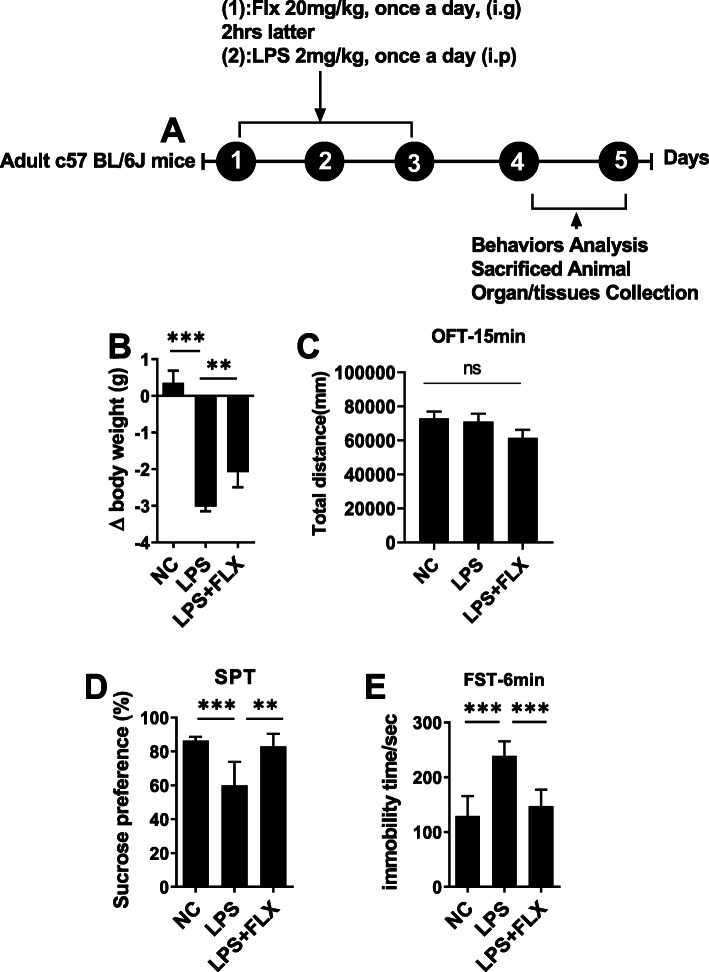
Fluoxetine reduced LPS-induced depressive-like behaviors. **a** Drug treatment schedule, **b** relative body weights, **c** open field test OFT, **d** forced swimming test, and **e** sucrose preference test. All the values are expressed as mean ± SEM: ANOVA followed by post hoc analysis. *p < 0.05, **p < 0.01, ***p < 0.001, ****p < 0.0001

In the first experiment, animals were divided into three groups (8–10 each group): saline-treated, lipopolysaccharide (LPS) (2 mg/kg/day, intraperitoneally), and LPS+ fluoxetine (20 mg/kg/day, orally). The drug treatment schedule has been shown in Fig. 1a. The LPS and fluoxetine dose were based on the previous study. After 24 h of the last LPS injection, mice were sacrificed after behaviors analysis (described in detail below). Serum and brain tissues were collected and stored at freezing temperatures (− 80 °C) until further investigation.

To explore the HDAC1 role in LPS-induced depression, a new experiment was planned, and mice were treated with an HDAC1 activator (exifone). The animal groups were saline-treated, LPS-treated, LPS+ fluoxetine+ exifone (54 mg/kg/day, i.p). The drug treatment schedule (Fig. 6a) was the same as above.

Further, to examine the association of HDAC1 and eERF2, a new experiment was designed, and mice were grouped: saline-treated, LPS-treated, LPS+ NH125 (1 mg/kg/day, i.p). The further drug treatment schedule (Fig. 8a) and behavior, as well as the organ collection process, were the same as above.

### Open field test (OFT)

To eliminate the animal sickness factors and avoid the biases due to sickness and blunted behaviors induced by LPS, OFT was performed according to the previously developed protocols [50]. Briefly, mice were adapted to the experimental room for 1 h and were placed in the chamber of 45× 45 × 30 cm. A total of a 15-min video was recorded to observed the mice locomotor activity. The total distance covered by mice was measured, analyzed, and expressed in meters.

### Sucrose preference test

A sucrose preference test was performed while using a two-bottle free-choice paradigm. Mice were habituated with a 1% sucrose solution for 3 days and finally grouped randomly. To assess the individual sucrose intake, mice were deprived of water and food for 24 h on the 3 days of drug administration. On the next day, each mouse had free access to two bottles containing sucrose and water, respectively. The position of water and sucrose-containing bottles were changed after 12 h. Finally, the volume of consumed water and sucrose solution were recorded and calculated by the following formula:

$$ \mathrm{SPT}=\frac{\mathrm{Sucrose}\ \mathrm{consumption}}{\mathrm{Water}\ \mathrm{and}\ \mathrm{sucrose}\ \mathrm{consumtion}}\times 100\% $$

### Forced swimming test (FST)

The forced swimming test (FST) was performed according to previously developed protocols [51]. The experimental animals were trained for swimming and pre-experiment FST was performed to select healthy and normal mice. To perform the FST, the animals were placed in a Plexiglas cylinder (height: 70 cm, diameter: 30 cm) filled with water over the 30 cm level at a temperature of 23 ± 1 °C. The video was taped for 6 min and the last 5 min were blindly analyzed. Mice were considered immobile when they remained floating motionless in the water and just making a move to keep their nose above the water surface. The horizontal movement of the animals throughout the cylinder was defined as swimming while vertical movement against the wall of the cylinder was defined as climbing. EthoVision XT was used to record the video and analysis.

### Tail suspension test (TST)

The tail suspension test was performed as described previously as [50, 52]. Briefly, the mice the upside down about 40 cm above the floor by placing adhesive tape 1 cm from the tail tip. The immobility time was scored for the first 2 min of the total 4-min video. EthoVision XT software was used for TST recording and analysis.

### BV2 cell line culture protocol and stimulation

Mouse microglial BV2 cell lines were grown in high glucose Dulbecco’s modified Eagle’s medium (DMEM) supplemented with 10% fetal bovine serum (FBS) (Gibco, Waltham, MA). The cells were maintained in a humidified incubator with 95% air and a 5% CO_2_ atmosphere at 37 °C. Medium containing the appropriate agents was replaced every other day. When the cells grew to a density of about 90%, exifone was added to the cell medium, fluoxetine was added to the cell culture after 16 h, lipopolysaccharide was added after 2 h, and cells were harvested after 4 h.

### Cell viability

Cell viability using cell counting kit-8 of MedChemExpress (Monmouth Junction, NJ, USA). Briefly, inoculate cell suspension (100 μL/well) in a 96-well plate. Add different concentration exifone to cell medium, pre-incubated the plate in a humidified incubator at 37 °C, 5% CO_2_. After 20 h, add 10 μL of the CCK-8 solution to each well of the plate. Then incubate the plate for 1–4 h in the incubator, measure the absorbance at 450 nm using a microplate reader.

### Short hairpin (sh)RNA expression constructs and treatment

shRNA plasmid coding for HDAC 1 was purchased from Haixing Biosciences (88 keling Road, Huqiu District, Suzhou, Jiangsu, China. The shRNA targeting HDAC1 had the sequence 5′-GCTGGAAAGGCAAGTATTATCGAGATAATACTTGCCTTTGCCAGC-3′. The scrambled RNA sequence, used as a control, had the sequence 5′-CCTAAGGTTAAGTCGCCCTCGCTCGAGCGAGGGCGACTTAACCTTAGG-3. The plasmid (2.5 μg/well) containing shRNA was transfected to BV2 cells. After 30 h, the BV2 cells were treated with LPS (100 ng/ml). Finally, after 4 h of LPS treatment, cells were collected and proceeded for further analysis.

### Nitric oxides and H_2_O_2_ measurement

The level of NO and H_2_O_2_ was analyzed by a commercially available kit (Beyotime Institute of Biotechnology, China, CAT# S0021M, and CAT# S0038, respectively) [53, 54] and measured the absorbance at 540 nm using a microplate reader (Biorad-Benchmark, USA).

### TBAR assay

TBARS level was estimated [55] to determine the damage to lipids caused by reactive oxygen species in various experimental groups. Briefly, 0.1 ml of sample, 0.1 ml FeSO_4_, 0.1 ml Tris-HCl, 0.6 ml distilled water, and 0.1 ml ascorbic acid were incubated at 37 °C in a test tube 15 min, and then 1 ml TCA and 2 ml TBA were added. These plugged test tubes were incubated for 15 min at 100 °C followed by centrifugation at 3000 rpm for 10 min. The supernatant O.D. was determined at 532 nm, and the following formula was applied to estimate TBARS as nM/mg protein: TBARS (nM/mg protein) = O.D × total volume × sample volume × 1.56 × 105 × mg protein/ml (1.56 × 105 = molar extinction coefficient).

### ELISA

The frozen hippocampal and cortical tissue was lysed with RIPA buffer and homogenized on ice. Supernatants were collected after centrifugation and stored at freezing temperature for further analysis. The expression of cytokines was quantified using ELISA kits (ABclonal) according to the manufacturer’s protocols. Briefly, after washing the wells of 96-well plate, 100 μL standard/sample was added and incubated for 2 h at 37 °C. The plate was then washed, and a biotin-conjugated antibody (1:30) was added to each well. The plate was incubated for 1 h at 37 °C. streptavidin-HRP was added for 30 min at 37 °C. Finally, the reaction was stopped and the optical density was measured accordingly.

### Immunofluorescence

Immunofluorescence staining was performed according to previously reported protocols [56]. Briefly, brain tissue sections (20-μm thick) were washed with PBS for 15 min (5 min × 3). After washing, the sections were treated with blocking buffer (10% goat serum in 0.3% Triton X-100 in PBS) for 1 h at room temperature. After blocking, the tissue was treated with primary antibodies (Iba1, GFAP) for overnight at 4̊ °C. The next day, secondary antibodies (Alexa Flour secondary antibodies, ThermoFisher) were applied at room temperature for 1 h. The sections were washed with PBS for 5 min three times. After washing, the sections were transferred to slides, and glass coverslips were mounted using the mounting medium. The images were taken under inverted fluorescence microscope I X73 Olympus.

### Golgi staining

The FD Rapid GolgiStain Kit (FD NeuroTechnologies, Ellicott City, MD) was used to perform Golgi staining. Briefly, after removing, the animal brain was rinse quickly in double distilled water, immersed impregnation solutions (A/B) (5 ml solution for each tissue), and store at room temperature for 2 weeks. The brain tissues were transferred to solution C and store for 72 h (the solution was replaced after 24 h), followed by freezing. After that, 100- to 200-μm sections were prepared using a sliding microtome and mount to gelatin-coated microscope slides. Then, the brain tissue was placed in staining solution for 10 min and rinsed with double distilled water, followed by dehydration (sequential rinse 50%, 75%, and 95% ethanol) and xylene treatment. Finally, examined under inverted fluorescence microscope IX73 Olympus.

### Western blotting

According to the developed protocols, western blotting was performed. Briefly, denatured samples (boiled at 100 °C for 10 min) were separated on SDS-PAGE and then transferred to the nitrocellulose membrane. The membrane was blocked in with non-fat milk in TBST (tris-buffered saline, 0.1% Tween 20), then incubated in primary antibody (1: 500; 1:1,000) (list of antibodies with dilution used, Suplementraty data), overnight at 4 °C. The next day, the membrane was treated with a secondary antibody (1:1000) for 1 h at 4 °C. For detection, the ECL super signal chemiluminescence kit was used according to the manufacturer’s protocol. Blots were developed using Chemidoc mp Bio-red. The densitometry analysis of the bands was performed using Image Lab Software.

### Statistical analysis

Western blot bands and morphological data were analyzed using ImageJ and Image Lab Software (Image J 1.30) and analyzed by SPSS Statistics 21 (IBM, US) and GraphPad Prism 8 software. Data were presented as mean ± SEM. Before analysis, data normality tests were performed for all behavior tests (Fig. S4). One-way ANOVA followed by post hoc Tukey’s multiple comparison tests were performed to compare different groups. *P* < 0.05 was regarded as significant. *p< 0.05, **p < 0.001, ***p < 0.0001, and ****p < 0.00001.

## Results

### Fluoxetine reduced LPS-induced depressive-like behavior

LPS is a well-established inflammatory agent and widely employed to induce depressive-like behavior [51, 57]. Herein, to study the antidepressant role of fluoxetine, we examined LPS-treated animals in validated paradigms, including body weight (Fig. 1b), open field test (Fig. 1c), immobility (Fig. 1d), and sucrose preference (Fig. 1e) for depression-like behaviors. As shown in Fig. 1, LPS-treated mice showed decreased body weight and sucrose preference of less than 65% for a 1% sucrose solution; however, after fluoxetine treatment, body weights, and sucrose preference were significantly recovered. In the forced swimming test, the immobility time was increased in LPS-treated mice, which was reversed by fluoxetine treatment.

### Fluoxetine abolished LPS-induced neuroinflammation

**Fig. 2:**
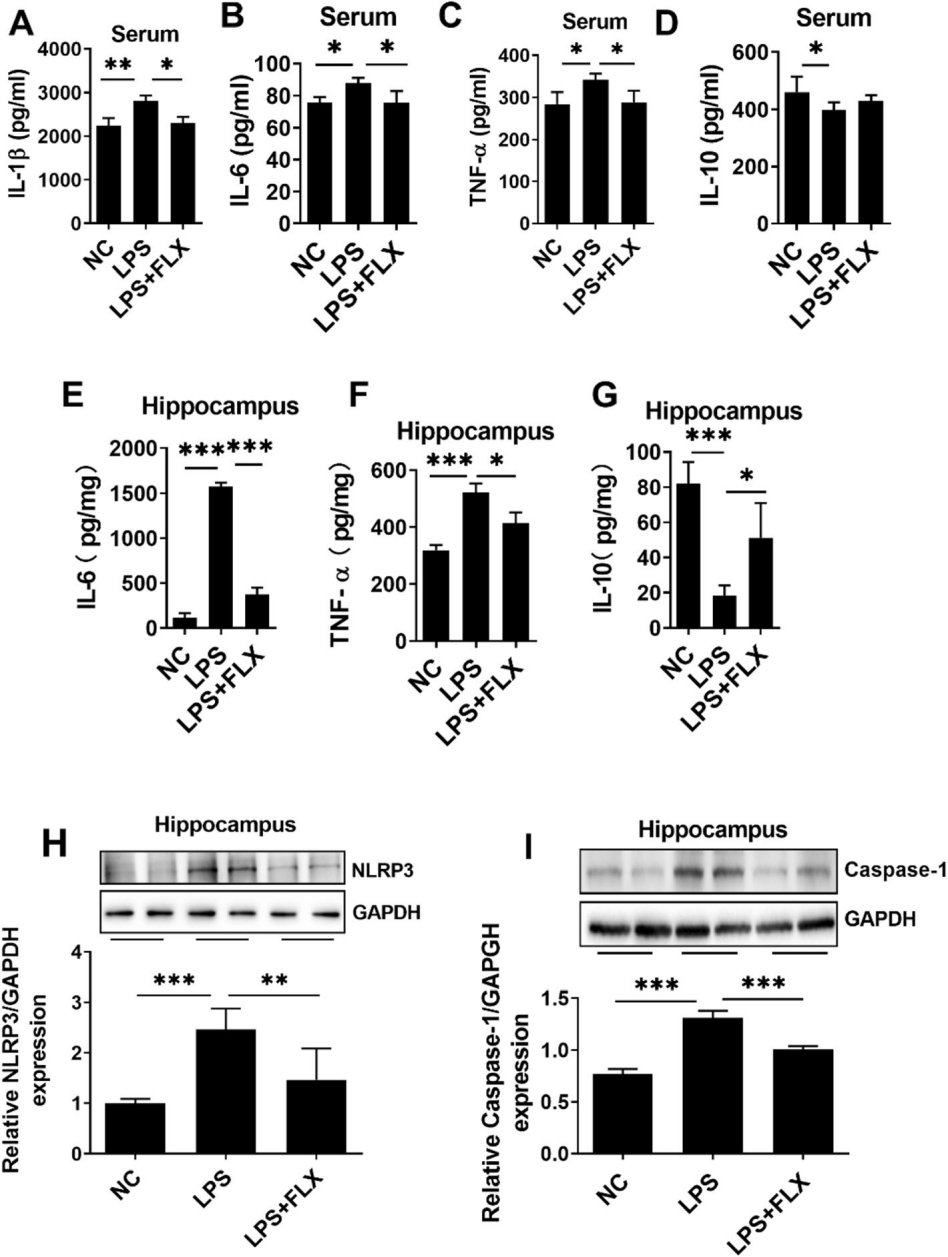
Fluoxetine reduced LPS-induced neuroinflammation. **a** Serum IL-1β**, b** serum IL-6, **c** serum TNF-α, **d** serum IL-10 level, **e** hippocampal IL-6, **f** hippocampal TNF-α, **g** hippocampal IL-10, **h** NLRP3 level column graph, and representative western blots for mice treated with LPS and fluoxetine. **i** Total level of caspase-1 and representative western blots. All the values were normalized with GAPDH. Image Lab Software was used for blots quantitative analysis and was analyzed via GraphPad prism. Data were expressed as ± SEM, one-way ANOVA followed by post hoc analysis. *p* = < 0.05 were considered significant. *p < 0.05, **p < 0.01, ***p < 0.001, ****p < 0.0001

**Fig. 3:**
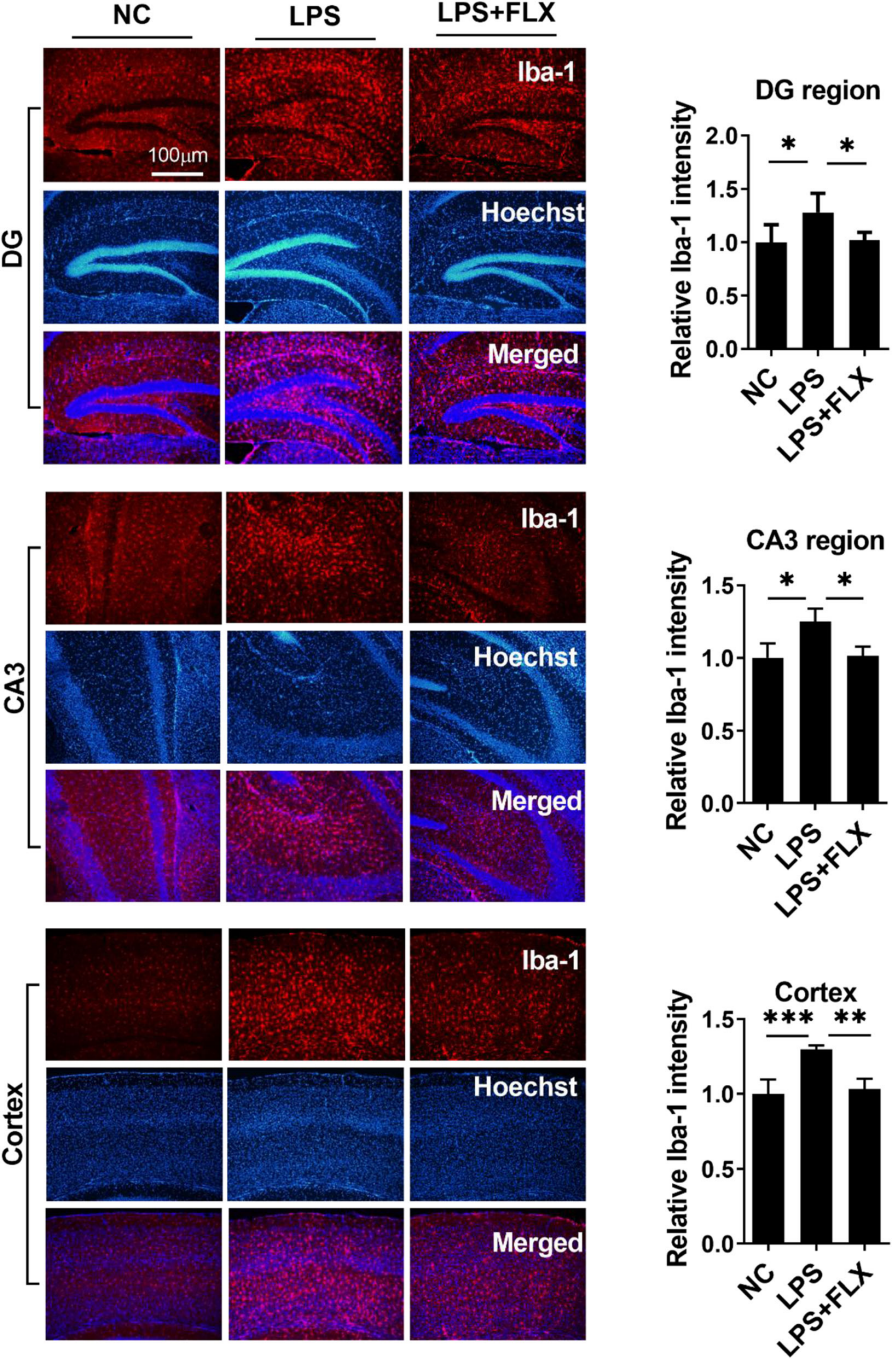
Fluoxetine reduced LPS effect on Iba-1 expression. Microscopy results of Iba-1 expression in the different experimental groups of brain tissues, with respective bar graphs (*n* = 6), x10 magnification. The image data were collected from three independent experiments and were analyzed by ImageJ software. The differences have been shown in the graphs. Data were expressed as ± SEM, one-way ANOVA followed by post hoc analysis. *p* = < 0.05 were considered significant. *p < 0.05, **p < 0.01), ***p < 0.001, ****p < 0.0001

**Fig. 4:**
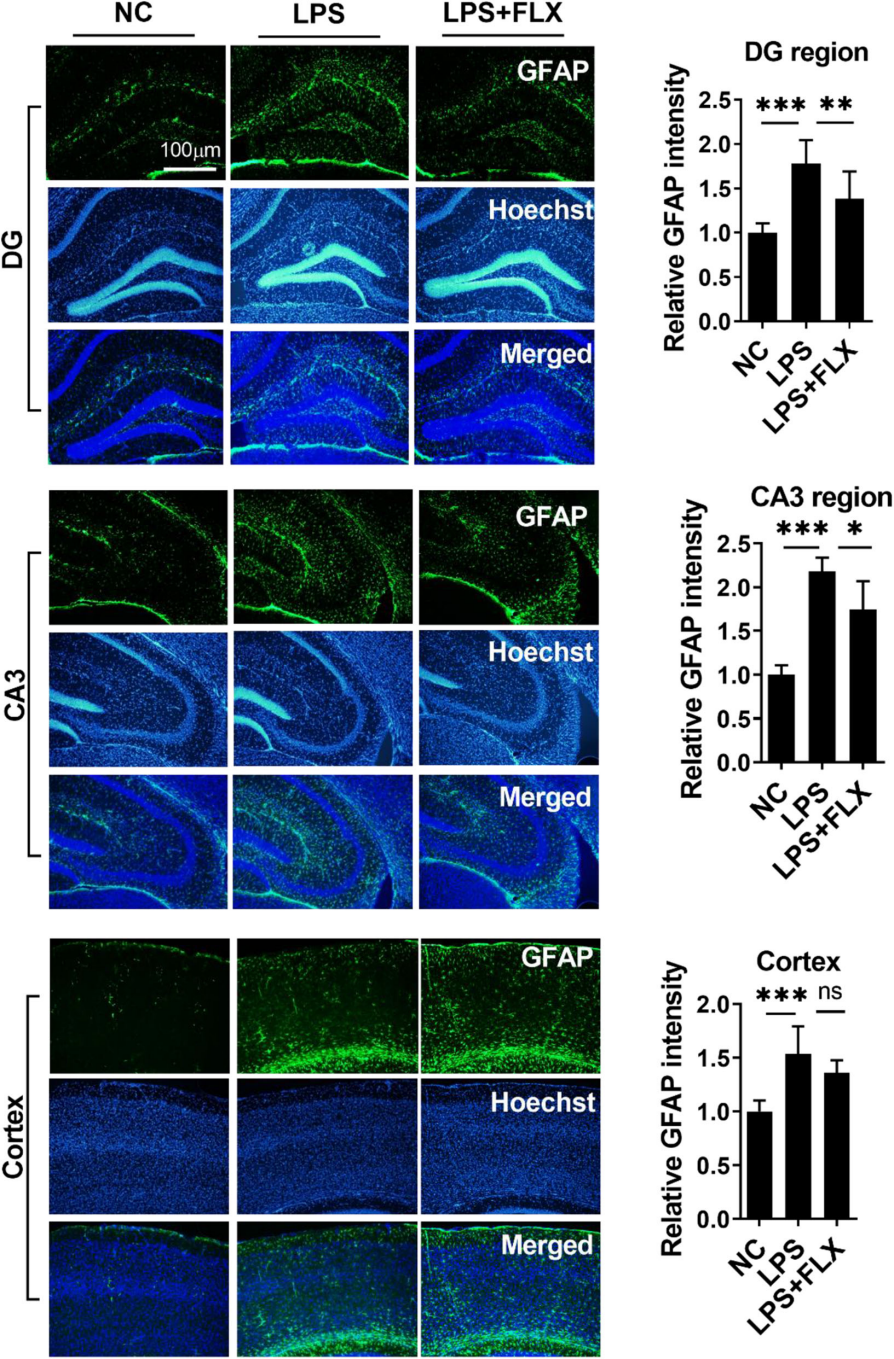
Fluoxetine reduced LPS effect on GFAP. Microscopy results of GFAP expression in the different experimental groups of brain tissues, with respective bar graphs (*n* = 7), x10 magnification. The image data were collected from three independent experiments and were analyzed by ImageJ software. The differences have been shown in the graphs. Data were expressed as ± SEM, one-way ANOVA followed by post hoc analysis. *p* = < 0.05 were considered significant. *p < 0.05, **p < 0.01, ***p < 0.001, ****p < 0.0001

We then evaluated the anti-inflammatory effects of fluoxetine. LPS administration enhanced the expression of pro-inflammatory cytokines including tumor necrosis factor (TNF-α), interleukin 1β (IL-1β), interleukin 6 (IL-6), and oxidative stress (Fig. S1) and reduced anti-inflammatory cytokine IL-10 level in the serum and hippocampus of the experimental animals (Fig. 2a–g). Further, NLR family pyrin domain-containing 3 (NLRP3)-mediated neuroinflammation plays a key role in neurological disorders including MDD [58]. LPS treatment significantly enhanced NLRP3 and caspase-1 expression in the hippocampus (Fig. 2h and i), which is responsible for the maturation of cytokine such as IL-β and finally leads to pyroptosis [59]. To further corroborate LPS-induced neuroinflammation, we measured ionized calcium-binding adaptor molecule 1 (Iba-1) and glial fibrillary acidic protein (GFAP) expression in the hippocampus and the prefrontal cortex regions (Figs. 3 and Fig. 4). Our immunofluorescence results indicated that LPS-treatment significantly enhanced Iba-1 and GFAP expression in DG and CA3 regions of the hippocampus, as well as in the prefrontal cortex region. Interestingly, fluoxetine treatment reversed the above LPS-induced changes including increased pro-inflammatory cytokines, decreased anti-inflammatory cytokine, increased NRLP3, caspase-1, Iba-1, and GFAP expression, suggesting the strong anti-inflammatory effects of fluoxetine.

### Fluoxetine improved LPS-dysregulated synaptogenic defects via HDAC1 and eEF2 regulation

**Fig. 5:**
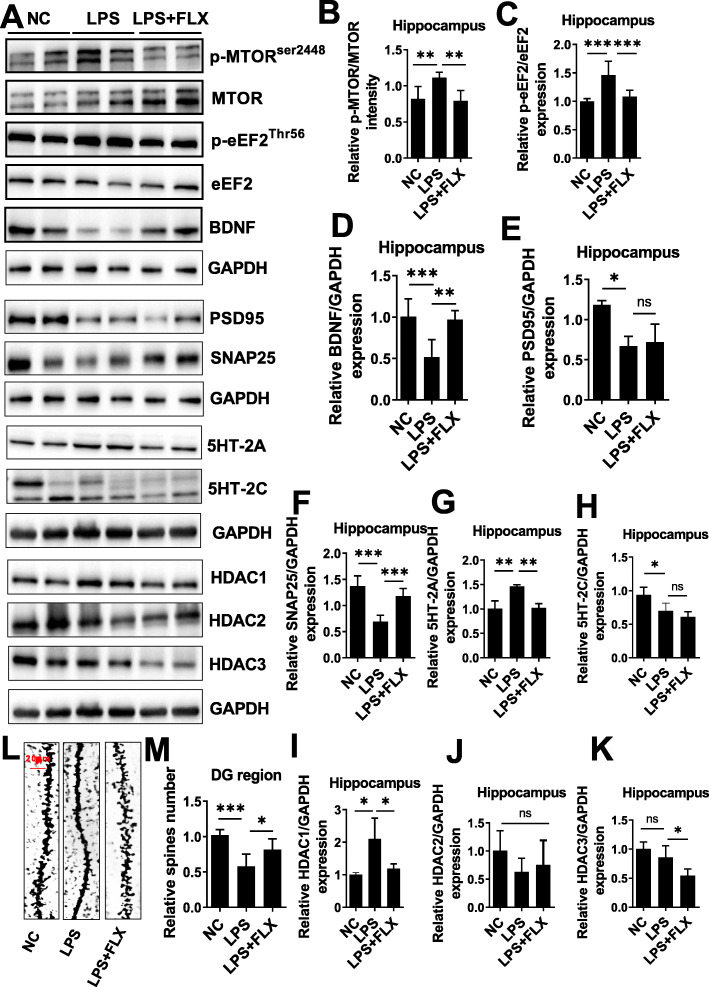
Fluoxetine attenuated LPS effect on mTOR/eEF2/BDNF/SNAP25/PSD95 and HDACs. **a** Representative immune blot images and average protein levels of **b** p-mTOR, **c** p-eEF2, **d** BDNF, **e** PSD95, **f** SNAP25, **g** 5HT2A, and **h** 5HT-2C. **i–k** Average level of HDAC1, HDAC2, and HDAC3 levels, respectively. **l**, **m** Golgi staining showing spine density and column graph showing spin numbers. Image Lab Software was used for blot quantitative analysis and was analyzed via GraphPad prism. Data were expressed as ± SEM, one-way ANOVA followed by post hoc analysis. *p* = < 0.05 were considered significant. *p < 0.05, **p < 0.01), ***p < 0.001, ****p < 0.0001

Dysregulated neurogenesis and synaptogenesis have been reported in the brain of patients with major depressive disorders and multiple molecular pathways are believed to be disrupted in these processes, including BDNF, TrkB, PSD95, and SNAP25 [30, 33, 39]^,^ [60]. Destabilized synaptogenesis has been detected during MDD along with declined BDNF and TrkB expression [61, 62]. Our results showed decreased BDNF, PSD95, and SNAP25 expression in LPS-treated mice hippocampus, while fluoxetine treatment markedly reversed these changes (Fig. 5a and d–f). Synaptogenesis-related signaling molecules such as Akt/mTOR were then examined as their activities are involved in various neurological disorders including MDD [63]. As shown in Fig. 5a, b, LPS-treatment increased mTOR phosphorylation that could be attenuated by fluoxetine administration. Similarly, as a key player in protein synthesis and possibly the core of depression, eEF2 activity and expression were then examined [30] [32, 33]. Enhanced eEF2 phosphorylation could be detected in LPS-treated mice hippocampus, which was diminished upon fluoxetine treatment (Fig. 5a, c).

To validate the dysregulated protein synthesis and eventually the synaptic morphological changes, spine numbers were measured and analyzed with Golgi staining. Significantly reduced spines numbers (Fig. 5l, m) were found in LPS-administrated mice as compared to fluoxetine-treated animals. Furthermore, serotonin receptor changes were investigated through which fluoxetine act as an antidepressant [64, 65]. Fluoxetine treatment significantly decreased LPS-mediated 5-hydroxytryptamine receptor (5HT-2A) and 5HT-2C expression in the mice hippocampus (Fig. a, g, and h). Besides, accumulating studies show dysregulated HDACs consequently lead to impaired acetylation and deacetylation in translational control [66], which could play a key role in the pathophysiological development of MDD [67]. We then measured HDAC1, 2, and 3 expressions in the hippocampal tissues of the experimental animals. LPS administration significantly enhanced HDAC1 expression but not HDAC2 and HDAC3 expression, which could be substantially attenuated by fluoxetine (Fig. 5a, i–k).

### Fluoxetine prevented neuroinflammation via HDAC1 inhibition

**Fig. 6:**
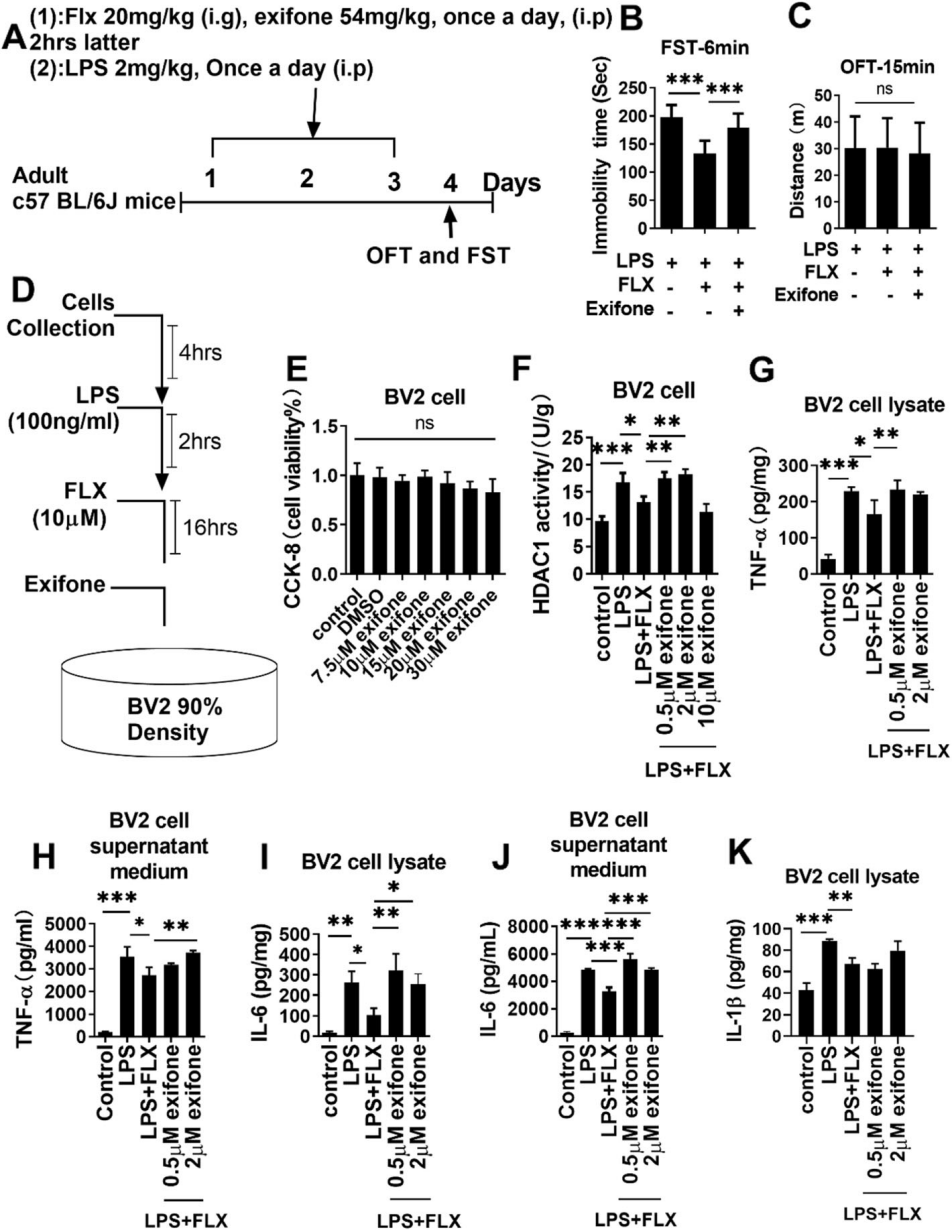
Exifone treatment reversed the neuroprotective effect of fluoxetine. **a** Drug treatment schedule, **b** FST, **c** OFT, **d** BV-2 cell drug treatment schedule, **e** BV-2 cell viability assay, **f** HDAC1 activity in exifone, LPS, and fluoxetine-treated BV-2 cells, **g** TNF-α level in cell lysate, **h** TNF-α in cell supernatant, **i** IL-6 level in cell lysate, **j** IL-6 in cell supernatant, **k** IL-1β level in exifone, LPS, and fluoxetine-treated BV-2 cell lysates. Data were expressed as ± SEM, one-way ANOVA followed by post hoc analysis. *p* = < 0.05 were considered significant. *p < 0.05, **p < 0.01), ***p < 0.001, ****p < 0.0001

To further delineate the role of HDAC1 in LPS-induced neuroinflammation allied depression and the antidepressive effects of fluoxetine, exifone (Fig. 6a), a potent HDAC1 activator [68], was employed. As shown in Fig. 6b, the antidepressive effect of fluoxetine was blocked by exifone as compared to LPS-treated mice, indicating the necessity of HDAC1 in fluoxetine’s antidepressant action.

In vitro analysis was performed to further explore the roles of HDAC1 in an inflammatory response and eEF2-related protein synthesis. BV-2 murine microglial cells [69] were treated with different concentrations of exifone (0.5, 2, and 10 μM) for 16 h and then with fluoxetine (100 ng) for 2 h, followed by LPS treatment (100 ng/ml) for 4 h (Fig. 6d). After confirming cell viability (Fig. 6e), HDAC1 expression was measured in collected BV-2 cells. Interestingly, exifone reversed the suppressed HDAC1 level by fluoxetine, with the most significant effects observed at 0.5 and 2 μM.

**Fig. 7:**
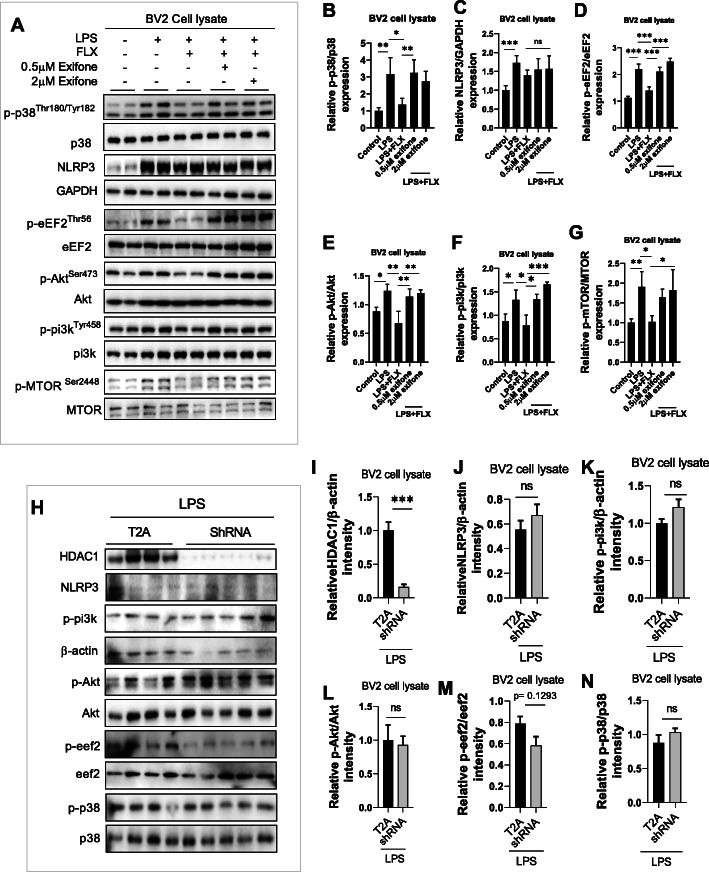
Exifone attenuated fluoxetine effects during in vitro analysis. **a** Representative immune blot images and average protein levels of **b** p-p38, **c** NLRP3, **d** p-eEF2, **e** p-Akt, **f** p-pi3k, and **g** p-mTOR.**h** Representative immune blot images and average protein levels of **i** HDAC1, **j** NLRP3, **k** p-pi3k, **l** p-Akt, **m** p-eeF2, and **n** p-p38. Image Lab Software was used for blot quantitative analysis and was analyzed via GraphPad prism. Data were expressed as ± SEM, one-way ANOVA followed by post hoc analysis. *p* = < 0.05 were considered significant. *p < 0.05, **p < 0.01), ***p < 0.001, ****p < 0.0001

Next, the contribution of HDAC1 in LPS-induced inflammatory response was evaluated in exifone treated BV-2 cells in the presence of LPS and/or fluoxetine. LPS treatment significantly increased pro-inflammatory cytokines, including TNF-α, IL-1β, and IL-6 expression, which could be markedly reversed by fluoxetine treatment. Interestingly, the anti-inflammatory effects of fluoxetine were tremendously abolished by exifone (Fig. 6h–k), suggesting the requirement of HDAC1 in fluoxetine’s anti-inflammatory response. Interestingly, the signaling specificity of exifone was further identified with NLRP3 and p38, two distinct yet integrated molecules contributing to inflammatory responses [58, 70, 71]. Our results showed the effects of exifone were selective in that it could reverse phosphorated p38 changes (Fig. 7a, b) but not NLRP3 expression affected by fluoxetine (Fig. 7a, c), demonstrating HDAC1 was not general anti-inflammatory signaling involved in fluoxetine.

To validate further the role of HAC1, the proteins, including NLRP3, p-p38, p-pi3k, p-Akt, and p-eEF2 level, were measured using immunoblot in BV2 cells transfected with HDAC1 shRNA in the presence of LPS (Fig. 7h). Surprisingly, we did not find any significant changes in the expression of these proteins (Fig. 7h-n); however, decrease levels of TNF-α and IL-6 were detected in shRNA-treated BV2 cells compared to control subjects (Fig. 2i, j). It indicates that HDAC1 could play a positive role in the progression of neuroinflammation and its associated pathologies.

### HDAC1-induced depressive-like behaviors were mediated by eEF2 inhibition

Previous studies suggest an association between neuroinflammation and dysregulated protein synthesis, leading to depressive-like behaviors [72]. Herein, protein synthesis regulatory factor eEF2 was measured in BV-2 cells. Exifone treatment abolished the reducing effects of fluoxetine on LPS-enhanced eEF2 phosphorylation (Fig. 7a, d). Similarly, it also diminished the reversing effects of fluoxetine on LPS-increased p-Akt/p-PI3k/p-mTOR expression (Fig. 7a, e–g), strongly supporting a pivotal role of HDAC1 in protein synthesis possibly via regulating mTOR/Akt/PI3k signaling and eEF2 activity, which may contribute to augmented synaptogenesis underlying the therapeutic mechanisms of fluoxetine.

**Fig. 8:**
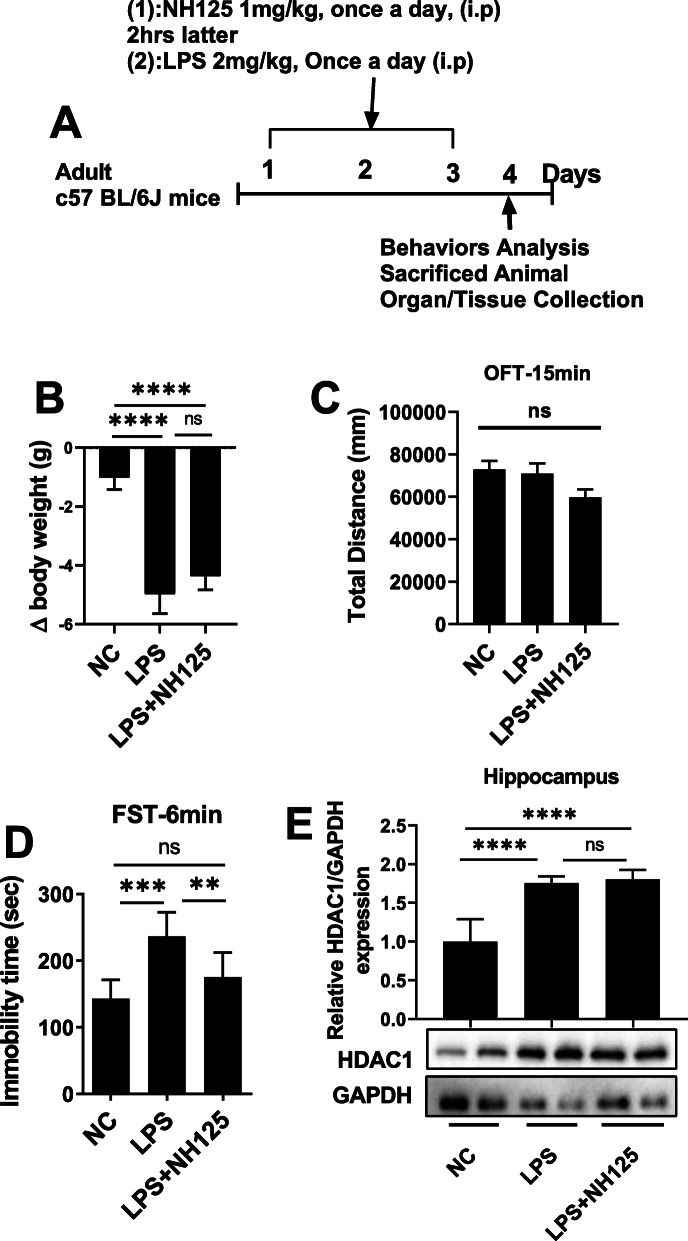
NH125 reduced LPS-induced changes. **a** Drug treatment schedule, **b** relative body weights, **c** OFT, **d** FST, **e** average protein level of HDAC1, and western blot image, normalized by GAPDH. Image Lab Software was used for blot quantitative analysis and was analyzed via GraphPad prism. Data were expressed as ± SEM, one-way ANOVA followed by post hoc analysis. *p* = < 0.05 were considered significant. *p < 0.05, **p < 0.01), ***p < 0.001, ****p < 0.0001

To further evaluate whether eEF2 is a downstream target of HDAC1, animals were treated with eEF2 kinase inhibitor NH125 (Fig. 8a) [73], which may reduce eEF2 phosphorylation. NH125 treatment reduced LPS-induced immobility (Fig. 8d). At the same time, it enhanced LPS-decreased sucrose preference (Fig. S2A). However, it did not significantly alter body weights (Fig. 8b), locomotor activity (Fig. 8c), and immobility time during TST (Fig. S2B). Further results indicated that NH125-treatment did not affect HDAC1 expression (Fig. 8d), confirming that eEF2 maybe the downstream target of HDAC1.

**Fig. 9:**
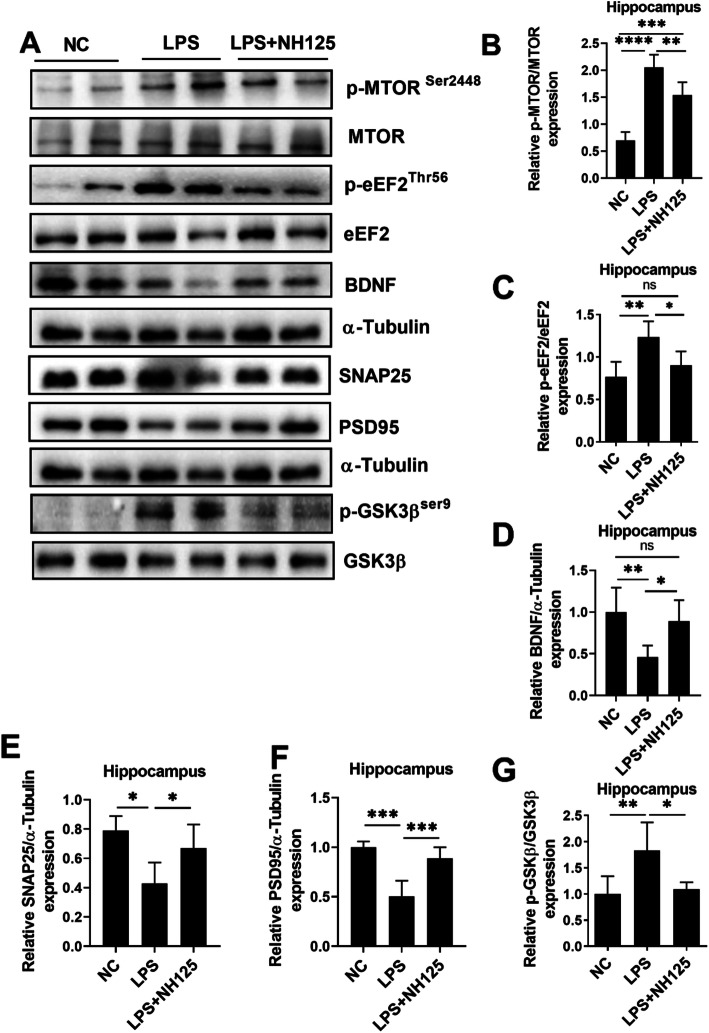
NH125 treatment attenuated LPS-induced changes in the brain of mice. **a** Representative immune blot images and average protein levels of **b** p-mTOR, **c** p-eEF2, **d** BDNF, **e** SNAP25, **f** PSD95, and **g** p-GSK3β. Image Lab Software was used for blot quantitative analysis and was analyzed via GraphPad prism. Data were expressed as ± SEM, one-way ANOVA followed by post hoc analysis. *p* = < 0.05 were considered significant. *p < 0.05, **p < 0.01), (***) *p* < 0.001, ****p < 0.0001

Activating mTOR rescues eEF2 from eEF2 kinase over-activating, which in turn upregulates BDNF [74, 75]. Next, we extend our investigation line to examine mTOR, BDNF, SNAP25, PSD95, and GSK3β changes after NH125 treatment. As shown in Fig. 9, NH125 treatment reduced the phosphorylation levels of eEF2 (Fig. 9a, c) and mTOR (Fig. 9a, b), while enhanced BDNF and eventually SNAP25 and PSD95 expression (Fig. 9a, d–f), suggesting an etiological role of eEF2 in LPS-induced synaptogenetic dysfunction and depression. GSK3β can activate eEF2 by reducing its phosphorylation, whereas inhibition of GSK3β can induce opposing effects [76]. Interestingly, our results also revealed that LPS treatment increased GSK3β phosphorylation and NH125 reversed the changes, indicating the possible involvement of GSK3β in eEF2 activation (Fig. 9a, g). Finally, the effects of NH125 on LPS-induced inflammation were examined to exclude its antidepressant effects via indirect anti-inflammation. Interestingly, NH125 treatment did not significantly affect LPS-altered Iba-1, IL-1β increase, GFAP, serum IL-1 β, IL-10, and IL-10 expression (Fig. S2; Fig. S3), suggesting the direct targeting of eEF2 on synaptogenesis processes.

## Discussion

Fluoxetine is one of the new generation antidepressants and the most commonly prescribed medicine for treating depression [77]. However, its efficacy and tolerability are controversial. Studies demonstrate variable and incomplete efficacy of fluoxetine, as 30–40% of depression patients do not show a significant response while 60–70% of patients do not experience remission [77–80]. Furthermore, previous results studying the molecular and cellular mechanisms of fluoxetine show ambiguous results of BDNF level and neurogenesis [78, 79], [80], [81–85], demanding further investigations to delineate the pharmacological basis of fluoxetine. Here, we demonstrated that apart from inhibiting neuroinflammation, fluoxetine could restore HDAC1-eEF2 activity (phosphorylation), which eventually reversed synaptogenic loss and depression-like phenotypes. These results not only for the first time, presented a novel antidepressant mechanism of fluoxetine but also offered an alternative choice to explore further therapeutic targets in depression.

Previous preclinical and clinical data support a strong association between neuroinflammation and depression [7, 9, 15, 74, 86]. Elevated cytokines are first recorded in patients with mood disorders, including MDD [87, 88]. Besides, the therapeutic effects of antidepressant and anti-inflammatory treatments against infection-induced sicknesses allied depressive-like symptoms further support the causal relation between neuroinflammation and depression [89–91]. HDACs play a critical role in immunity and regulate pleural TLR-targeted gene expressions [25, 92]. Although HDACs inhibition reduces inflammatory responses evoked by inflammatory agents like LPS [93], and altered HDACs including HDAC1 expression has been reported in the brain of patients with MDD [94, 95], the exact roles of HDACs in neuroinflammation and depression are still largely unknown. Elucidation of the etiological contribution of specific HDACs is essential for the development of novel therapeutic targets. Our findings of the anti-inflammatory activity of fluoxetine and its blockade by HDAC1 activator exifone strongly suggested HDAC1 in LPS-induced neuroinflammation. Additionally, exifone-induced alterations in mTOR/Akt/PI3k signaling and eEF2 activity further supported HDAC1 in the synaptogenesis associated with neuroinflammation-induced depression. It is yet unknown how HDAC1 modulated the transcription and translation processes. Previous studies examining the antidepressant effects of HDAC inhibitors suggest molecular adaptation in the brain, which might be due to chromatin remodeling by HDACs [29, 48, 49, 96]. Indeed, HDAC-induced acetylation is long proposed as a promising target for the novel treatment of psychiatric disorders, including MDD [24, 97]. Our findings supported a significant role of HDAC1 signaling in the pathophysiology of neuroinflammation-related depression. Further, a substantial role of HDAC1 has also been reported, as HDAC1 inhibitor treatment removes acetyl groups from histone, resulting in improved symptoms in different inflammatory diseases [98–100]. Herein, after blocking HDAC1 via shRNA, we did not detect any significant changes in the expression of genes, including NLPR3, p-p38, p-pi3k, and eEF2 involved in the molecular mechanism of neuroinflammation and its associated pathologies. Thus, it indicates that HDAC1 could play a positive role in accelerating neuroinflammation under LPS-induced stress conditions.

Dysregulated protein synthesis can play a crucial role in reduced synaptogenesis in the response of diverse stimuli, leading to depression. eEF2 contributes a significant part to the translational control of protein synthesis. Our results showed that LPS reduced eEF2 activity in the hippocampus and cortex, which could be reversed by fluoxetine through HDAC1. eEF2 is one of the downstream signaling molecules of mTOR and can be activated by eEF2K suppression [101]. Besides, mTOR serves as a kinase hub that can be activated by neurotransmitters and growth factors via PI3K/Akt signaling [102] and regulates post-synaptic protein translation to influence synaptogenesis [103].

Furthermore, eEF2 signaling regulates BDNF/TrkB protein synthesis, leading to BNDF suppression followed by depressive-like behaviors [61, 75, 104]. Accordingly, in our study, LPS-administration enhanced mTOR phosphorylation and altered synaptogenesis as demonstrated by reduced BDNF, PSD95, SNAP25, and spine numbers, which can also be abolished by fluoxetine treatment. In agreement with our findings, HDAC1 activity can be inhibited via PI3K/Akt signaling activation which might be dependent on GSK3β activity [105–108].

Disturbed protein synthesis and dysregulated synaptogenesis contribute to the pathogenesis of depression, as chronic stress can lead to synaptogenesis disruption [75, 89, 104, 109, 110], and fewer synapses and decreased synaptic protein have been reported in the brain of a patient with MDD [111, 112]. Antidepressants including SSRI (fluoxetine) trigger protein synthesis to increase dendritic growth and branching along with synaptic markers of PSD95 and synaptophysin in mTOR independent manner [102, 111]. Besides, disrupted BDNF/TrkB signaling under stress conditions causes a reduction of ERK/Akt signaling [109], which then influences synaptic maturation and stability via protein synthesis regulation [109, 113]. Our findings showed that NH125 disinhibited eEF2 (reduced phosphorylation) via suppression of eEF2 kinases increased BDNF, SNAP25, and PSD95 expression, as well as GSK3β activity in the hippocampus of the brain. These results suggested that disturbed signalings may cause synapse shrank and underline the pathological basis of depression, while strategies augmenting new spine formation would attenuate MDD-associated symptoms.

## Conclusion

In conclusion, our results demonstrated that increased HDAC1 expression contributed to neuroinflammation associated with MDD via inhibition of eEF2 activity and associated synaptogenesis. Fluoxetine could reverse these effects via increasing HDAC1-eEF2 activity and synaptogenesis, which ultimately abolished depressive-like symptoms besides its anti-inflammatory effects.

## Supplementary Information

**Additional file 1.**

## Data Availability

All data generated or analyzed during this study are included in this published article [and its supplementary information files].

## Abbreviations

HDACs: Histone deacetylases
LPS: Lipopolysaccharides
CNS: Central nervous system
MDD: Major depressive disorder
ROS: Reactive oxygen species
AKT: Serine-threonine protein kinase
GSK3: Glycogen synthase kinase 3
PIK3: Phosphatidylinositol 3-kinase
NF-κB: Nuclear Factor Kappa B Subunit
BDNF: Brain-derived neurotrophic factors
TrkB: Tropomyosin receptor kinase B
mTOR: Mammalian target of rapamycin
MAPK: Mitogen-Activated Protein Kinase
TLR4: Toll-like receptor 4
IL-1β: Interleukin 1 beta
IL-6: Interleukin 6
TNF-α: Tumor Necrosis Factor-alpha
FST: Force swimming test
TBARs: Thiobarbituric Acid Reactive Substance
ELISA: Enzyme-linked immunosorbent assay
GFAP: Glial Fibrillary Acidic Protein
IBA1: Allograft inflammatory factor 1
SNAP25: Synaptosome Associated Protein 25
PSD-95: Post-synaptic density protein 95
NLRP3: NLR family pyrin domain containing 3
EEF2: Eukaryotic elongation factor 2
DG: Dentate gyrus
CA3: Cornu Ammonis
ERK: Signal-regulated kinase
5HT: 5-Hydroxytryptamine
NH125: 1-Benzyl-3-cetyl-2-methylimidazolium Iodide
ANOVA: Analysis of variance
