# Correction: Fluoxetine regulates eEF2 activity (phosphorylation) via HDAC1 inhibitory mechanism in an LPS-induced mouse model of depression

**DOI:** 10.1186/s12974-022-02649-x

**Published:** 2022-12-29

**Authors:** Weifen Li, Tahir Ali, Chengyou Zheng, Zizhen Liu, Kaiwu He, Fawad Ali Shah, Qingguo Ren, Shafiq Ur Rahman, Ningning Li, Zhi-Jian Yu, Shupeng Li

**Affiliations:** 1 State Key Laboratory of Oncogenomics, School of Chemical Biology and Biotechnology, Peking University Shenzhen Graduate School, Shenzhen, 518055 China; 2 Riphah Institute of Pharmaceutical Sciences, Riphah International University Islamabad, Islamabad, Pakistan; 3 Department of Neurology, Affiliated ZhongDa Hospital, School of Medicine, Southeast University, Nanjing, China; 4 Department of Pharmacy, Shaheed Benazir Bhutto University, Sheringal, Dir, 18000 Pakistan; 5 Tomas Lindahl Nobel Laureate Laboratory, Precision Medicine Research Centre, The Seventh Affiliated Hospital of Sun Yat-Sen University, Shenzhen, 518107 China; 6 Department of Infectious Diseases and Shenzhen Key Laboratory for Endogenous Infections, The 6Th Affiliated Hospital of Shenzhen University Health Science Center, No. 89, Taoyuan Road, Nanshan District, Shenzhen, 518052 China; 7 Campbell Research Institute, Centre for Addiction and Mental Health, Toronto, ON Canada; 8 Department of Psychiatry, University of Toronto, Toronto, ON Canada

**Correction** **:** **Journal of Neuroinflammation (2021) 18:38** https://doi.org/10.1186/s12974-021-02091-5

Following publication of the original article [1], the authors identified an error in Fig. 5. The correct version of figure is given below.

**Fig. 5:**
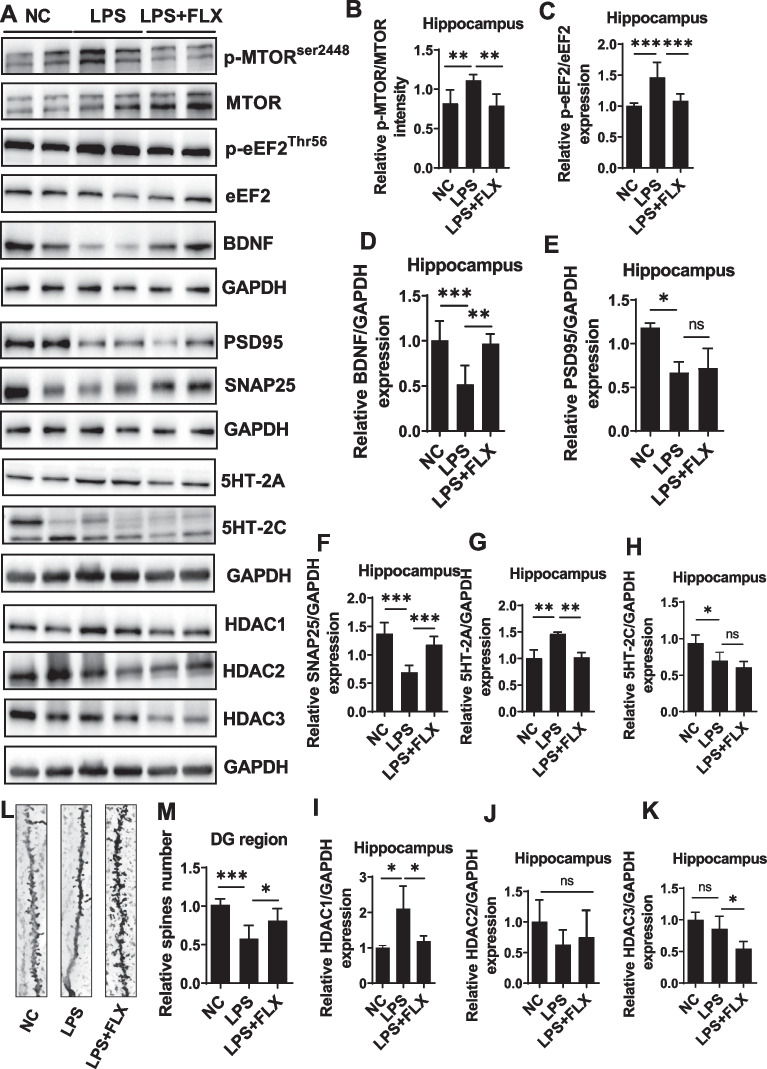
Fluoxetine attenuated LPS effect on mTOR/eEF2/BDNF/SNAP25/PSD95 and HDACs. **a** Representative immune blot images and average protein levels of **b** p-mTOR, **c** p-eEF2, **d** BDNF, **e** PSD95, **f** SNAP25, **g** 5HT2A, and **h** 5HT-2C. **i**–**k** Average level of HDAC1, HDAC2, and HDAC3 levels, respectively. **l**, **m** Golgi staining showing spine density and column graph showing spin numbers. Image Lab Software was used for blot quantitative analysis and was analyzed via GraphPad prism. Data were expressed as ± SEM, one-way ANOVA followed by post hoc analysis. p ≤ 0.05 were considered significant. *p < 0.05, **p < 0.01), ***p < 0.001, ****p < 0.0001

The original article has been corrected.
