# Supplementary material for: Fluoxetine regulates eEF2 activity (phosphorylation) via HDAC1 inhibitory mechanism in an LPS-induced mouse model of depression
Source: J Neuroinflammation. 2021 Feb 1;18:38. doi: 10.1186/s12974-021-02091-5 (PMC7852137; doi:10.1186/s12974-021-02091-5)
Supplement: Supplementary file 1 — Additional file 1. [file 12974_2021_2091_MOESM1_ESM.pdf]

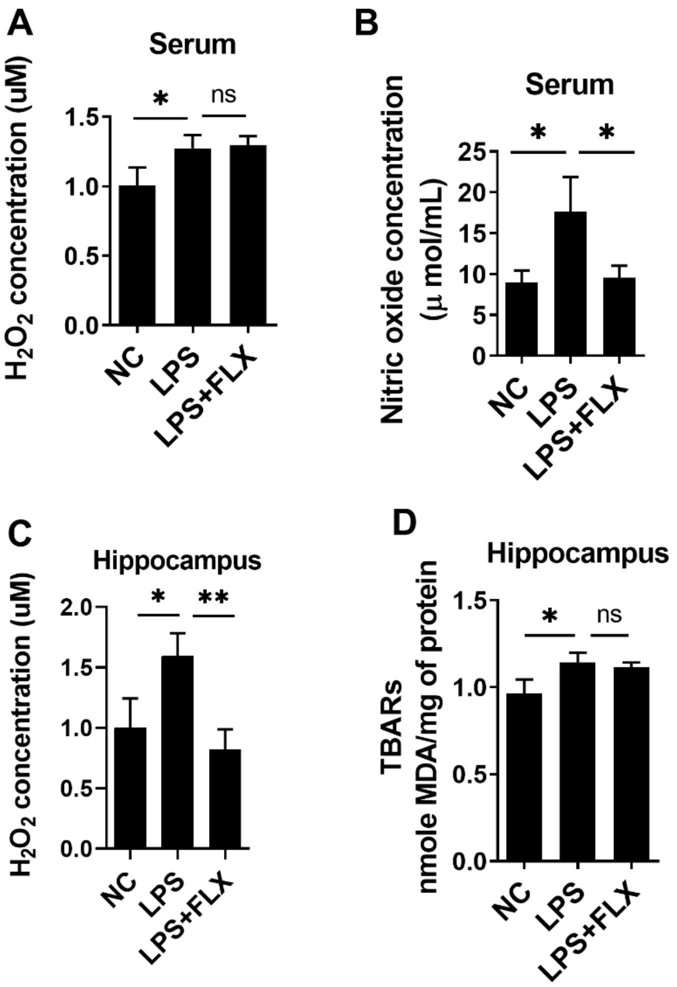

Figure S1: Fluoxetine reduced LPS-induced oxidative stress. A: Serum H2O2 concentration, **B:** Serum NO concentration, **C:** H2O2 concentration at hippocampus, D: TBARs level of hippocampus tissues. Data were expressed as ± SEM, One-way ANOVA followed by post-hoc analysis. p= <0.05 were considered significant. (*): p <0.05, (**): p <0.01), (***): p <0.001.

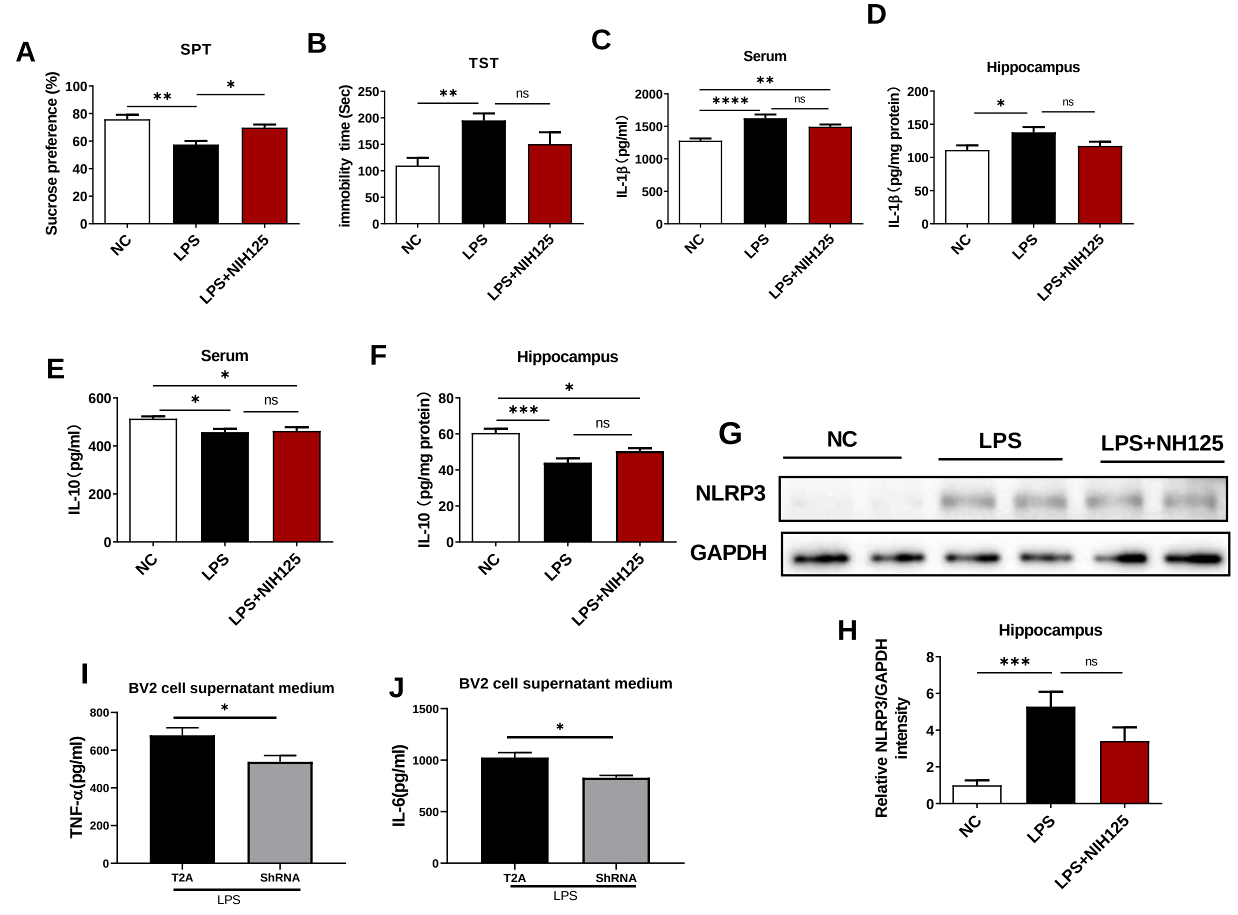

Figure S2: NH125 reduced LPS-induced behavior changes and neuroinflammation. A: SPT, **B:** TST, **C:** Serum IL-1β level, D: IL-1β level in the hippocampus, **E:** Serum IL-10, **F:** IL-10 level in the hippocampus, G and **H:** immune blot image showing NLRP3 expression and column graph showing the average level of NLRP3, normalized by GAPDH. **I and J:** TNF-α and IL-6 level in the shRNA treated BV2 cells. Image lab software was used for blots quantitative analysis and were analyzed via GraphPad prism. Data were expressed as ± SEM, One-way ANOVA followed by post-hoc analysis. p= <0.05 were considered significant. (*): p <0.05, (**): p <0.01), (***): p <0.001.

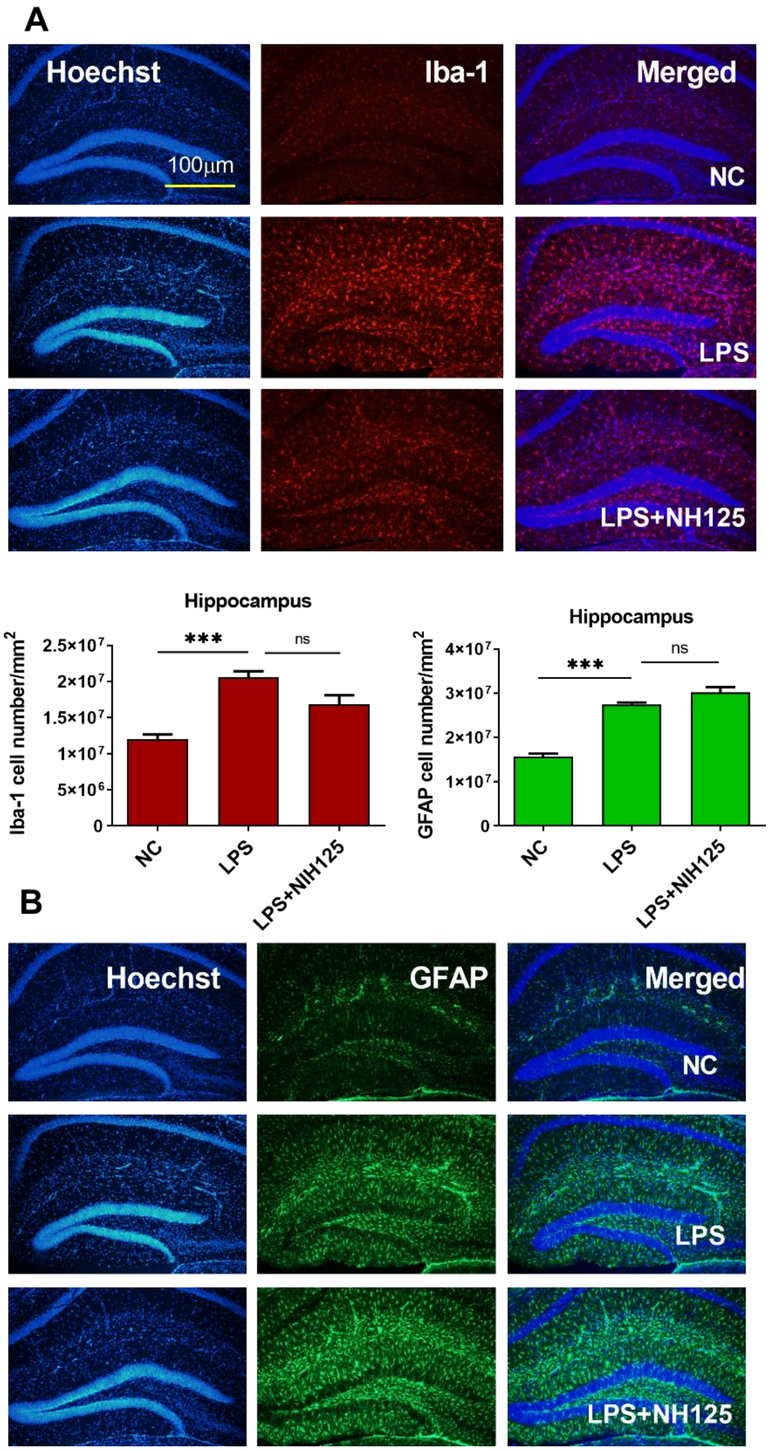

Figure S3: NH125 reduced LPS effect on glial cells activation: (A); Microscopy results of Iba-1 expression in the different experimental groups of brain tissues, with respective bar graphs. (B): Microscopy results of GFAP expression in the different experimental groups of brain tissues, with respective bar graphs. Magnification 10X. The image data were collected from three independent experiments and were analyzed by ImageJ software. The differences have been shown in the graphs. Data were expressed as ±

SEM, One-way ANOVA followed by post-hoc analysis. p= <0.05 were considered significant. (*): p <0.05, (**): p <0.01), (***): p <0.001.

A

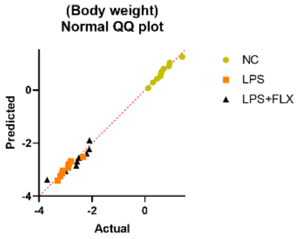

	NC	LPS	LPS+FLX
Test for normal distribution			
Anderson-Darling test			
A2*	0.2478	0.4619	0.6139
P value	0.6712	0.2006	0.0749
Passed normality test (alpha=0.05)?	Yes	Yes	Yes
P value summary	ns	ns	ns

B

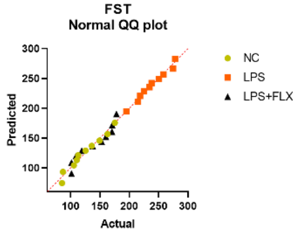

	NC	LPS	LPS+FLX
Test for normal distribution			
Anderson-Darling test			
A2*	0.2085	0.1600	0.4533
P value	0.8105	0.9238	0.2117
Passed normality test (alpha=0.05)?	Yes	Yes	Yes
P value summary	ns	ns	ns
D'Agostino & Pearson test			
K2	0.7452	0.2566	3.911
P value	0.6889	0.8796	0.1415
Passed normality test (alpha=0.05)?	Yes	Yes	Yes
P value summary	ns	ns	ns

C

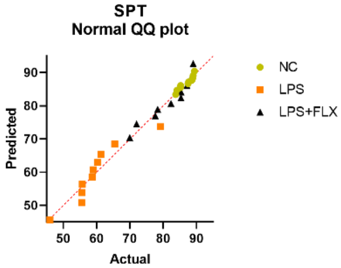

	NC	LPS	LPS+FLX
Test for normal distribution			
Anderson-Darling test			
A2*	0.5190	0.5975	0.4096
P value	0.1398	0.0870	0.2767
Passed normality test (alpha=0.05)?	Yes	Yes	Yes
P value summary	ns	ns	ns

Figure 4: Normality analysis for BW, FST and SPT tests.
